# Modified Geometry of ^106^Ru Asymmetric Eye Plaques to Improve Dosimetric Calculations in Ophthalmic Brachytherapy

**DOI:** 10.3390/jpm12050723

**Published:** 2022-04-29

**Authors:** Héctor Miras, José Antonio Terrón, Alejandro Bertolet, Antonio Leal

**Affiliations:** 1 Department of Medical Physics, Hospital Universitario Virgen Macarena, E-41009 Seville, Spain; jose.terron.sspa@juntadeandalucia.es (J.A.T.); alplaza@us.es (A.L.); 2 Instituto de Biomedicina de Sevilla, IBIS, E-41013 Seville, Spain; 3 Department of Radiation Oncology, Massachusetts General Hospital and Harvard Medical School, Boston, MA 02114, USA; abertoletreina@mgh.harvard.edu; 4 Departamento de Fisiología Médica y Biofísica, Universidad de Sevilla, E-41009 Seville, Spain

**Keywords:** ruthenium plaque, ophthalmic brachytherapy, Monte Carlo

## Abstract

Ru/Rh asymmetric plaques for ophthalmic brachytherapy have special geometric designs with a cutout intended to prevent irradiation of critical ocular structures proximal to the tumor. In this work, we present new geometric models for PENELOPE+PenEasy Monte Carlo simulations of these applicators, differing from the vendor-reported geometry, that better match their real geometry to assess their dosimetric impact. Simulation results were benchmarked to experimental dosimetric data from radiochromic film measurements, data provided by the manufacturer in the calibration certificates, and other experimental results published in the literature, obtaining, in all cases, better agreement with the modified geometries. The clinical impact of the new geometric models was evaluated by simulating real clinical cases using patient-specific eye models. The cases calculated using the modified geometries presented higher doses to the critical structures proximal to the cutout region. The modified geometric models presented in this work provide a more accurate representation of the asymmetric plaques, greatly improving the agreement between Monte Carlo calculations and experimental measurements. Lack of consideration of accurate geometric models has been shown to be translated into notable increases in dose to organs at risk in clinical cases.

## 1. Introduction

Uveal melanoma is a rare (two to eight cases per million in Europe) but aggressive disease. This tumor is the most common primary ocular tumor in adults and represents 83% of intraocular malignancies and around 60% of all non-skin melanomas. Uveal melanoma is mainly located in choroid (90%), although it could appear in ciliary body (6%) and iris (4%). It is an aggressive malignancy with high mortality rates (up to 90% at 15 years in posterior uveal melanoma) [1]. Treatment options for uveal melanomas are resection, radiation therapy and enucleation. Classically, enucleation was the main treatment to achieve local control, although, currently, plaque brachytherapy is the most commonly used treatment for small and medium melanomas, with local control rates of 88–98% at five years [2]. Results are equivalent to enucleation in terms of metastasis free survival and overall survival [3], while having additional benefits, such as better cosmetic results and vision preservation in most of the cases.

Plaque brachytherapy for uveal melanoma is a personalized treatment depending on tumor size and intraocular localization. Several isotopes (I-125, Ru-106, Pd-103, Co-60) are used, with beta-emitting Ru/Rh-106 ophthalmic applicators [4] being one of the preferred options to treat intraocular malignancies [5]. Historically, dose calculations in ocular treatments with Ru applicators (manufactured only by Eckert and Ziegler BEBIG, GmbH, Berlin, Germany) used to be only based on water and limited to the central axis of the plaque. The latest recommendations on ocular plaque therapy [6] emphasize the importance of a transition to model-based calculation algorithms [7], which allow personalized treatment planning. However, accurate dosimetry in these treatments is hindered by a number of special features, such as the small dimensions and complex shapes of the applicators, made of non-water equivalent materials, and the short range of beta particles, which produce steep-gradient dose distributions. All these factors challenge dose determination by means of both experimental measurements and Monte Carlo (MC) simulations. The dosimetry problem is even more pronounced for the special case of the asymmetric plaques, which have a cutout, so-called notch, to prevent irradiation of critical structures such as the optic nerve, the macula, or the lens. These hurdles likely contribute to the lack of abundant literature on asymmetric Ru plaque dosimetry and to the reported disagreements between calculations and experimental measurements or between different articles.

To the best of our knowledge, only three works [8,9,10] included some experimental results for asymmetric Ru plaques. All these works used in-house developed phantoms for the radiochromic film-based dosimetry. Taccini et al. [8] and Trichter et al. [10] presented dosimetric results in the form of isodose distributions and lateral profiles for the BEBIG asymmetric plaque models CIB and CIA, respectively, without comparing to other published data or calculations. Heileman et al. [9] performed film measurements of the asymmetric COB plaque dose, showing important discrepancies with respect to simulations with the MC code MCNP6, especially in the cut-out region.

Solc et al. [11] were one of the first to present MC results for an asymmetric applicator. They used MCNPX [12] to simulate the COB applicator and compared their results with both central axis (CAX) reference data provided by the manufacturer and off-axis surface point dose values specified on the calibration certificates. They reported large discrepancies for the off-axis surface points that were attributed to a lack of uniformity in the activity distribution across the active layer.

The most complete dosimetric dataset of Ru ophthalmic applicators was published by Hermida et al. [13], who used penEasy+PENELOPE [14,15] MC codes to simulate a set of different applicators, including the asymmetric types CIA and CIB. Their results were compared with the manufacturer’s reference data and other previously published results, obtained either from experimental measurements or MC simulations. They found good agreement for all plaques compared to manufacturer CAX reference data, but with large discrepancies with respect to the Taccini et al. [8] dose plane for the CIB plaque.

Of note, most of these published works only present partial dosimetric results for a single type of notched plaque, and all the works that present MC simulations using asymmetric plaques obtain great discrepancies compared to experimental data.

We recently published a paper [16] that presents *EyeMC*, an MC calculation system for ophthalmic brachytherapy treatments, either with COMS plaques loaded with I-125 seeds or with Ru applicators. In that work, we validated the MC models of the Ru plaques against data from the work by Hermida-López et al. [13] and the manufacturer’s reference data for the CAX using the penEasy+PENELOPE MC codes. While we found a general good agreement with Hermida’s database, there were significant differences in the lateral dose profiles for asymmetric Ru plaques. We also benchmarked our results against Plaque Simulator [17,18], the only tool commercially available for personalized ophthalmic brachytherapy treatments, obtaining similar deviations for asymmetric-type plaques as well.

In this work, we attempted to bridge the observed discrepancies by developing more accurate and detailed geometries for MC simulations. We used graphical mathematical tools to determine new values for the geometric parameters, which were then used to modify the geometry of the MC models of the asymmetric Ru plaques presented in our previous work [16]. These new models were benchmarked using experimental dosimetric data from (i) radiochromic film measurements, (ii) data provided by the manufacturer in the calibration certificates, and (iii) experimental results published in some of the works cited above. We expect the conclusions presented in this work will help to improve ophthalmic treatments with Ru plaques as a consequence of a more accurate determination of dose delivered to the tumor and to the critical structures in each patient.

## 2. Materials and Methods

### 2.1. Geometric Parameters

**Figure 1:**
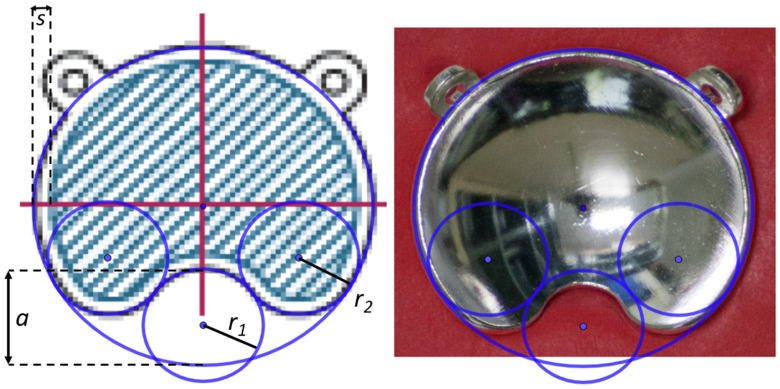
Images generated with GeoGebra of the COB plaque type with the superposition of the geometric forms that characterize the cutout using the parameter values given by the manufacturer (*a*, *r*_1_, and *r*_2_). The size of the inactive rim is represented by the parameter *s*. (**Left**) Plaque diagram extracted from the user manual, where the hatched area represents the active region. (**Right**) Image of a photograph taken for this work.

The only source of geometric information available for the notched plaques is provided by the manufacturer in the user manual of Ru applicators [19], where the geometry of the cutout is characterized by three parameters: Notch depth (*a*), notch interior radius (*r*_1_), and curvature radius of the edges (*r*_2_). In our previous work, we presented a graphical representation of these geometric parameters and their values tabulated for different asymmetric plaque types (Figure 3 and Table 1 from Miras et al. [16]). The size of the inactive rim, represented by parameter *s* in Figure 1, is another parameter not related to plaque geometry but that plays an important role in the lateral dose distributions, especially in the cutout region. The inner layer containing the active material does not extend to the edges of the plaque. By contrast, according to the information provided by the manufacturer, it is only extended up to 0.75 mm from the shell rim. Of all the mentioned works presenting calculations of the asymmetric plaques, only Solc et al. [11], Hermida-López et al. [13], and Miras et al. [16] included the Ru applicator user manual as a reference for the geometric information.

From visual inspection, we observed geometric differences in the cutout shape with respect to the diagrams of the plaques depicted in the user manual [19] and in ICRU 72 [4] (Figure 2.3 in ICRU 72). An example of this is shown in Figure 1. In order to verify this geometric characterization, we took photographs of the notched plaques available at our institution, the CIA, CIB, COB, and COC applicator models, obtaining high-quality images of the concave side of the plaques. By means of the software GeoGebra [20]—an interactive geometry, algebra, statistics, and calculus application—the different geometric forms that characterize the geometry of the asymmetric plaques were superimposed to the image of each plaque, redefining the optimal geometry to reproduce our real plaques. These geometric forms are an external circle with the diameter of the plaque, a small interior circle that defines the cutout, and two symmetric circles tangent to both the cutout and external circles that define the curvature of the notch edges.

### 2.2. Surface Dose Reference Data

With each ophthalmic applicator, the manufacturer provides a calibration certificate containing absolute dose rate values at 11 CAX points and relative surface dose rate values at 33 surface points normalized to the CAX point at 1 mm depth. These values are obtained from point measurements using a plastic scintillator detector with a diameter of 1.0 mm and height of 0.5 mm. Up to 2019, the calibration was traceable to the primary standard of the National Institute of Standards and Technology (NIST), and the reported uncertainty was +/− 20% (k = 2). Since 2020 the calibration has been traceable to the primary standard of the University of Wisconsin Radiation Calibration Lab with a +/− 11% uncertainty.

In addition, the user manual [19] of the Ru ophthalmic applicators provides reference relative dose rate values for all plaque types in the CAX obtained from the average measurements from different plaques for each applicator type. However, no reference data is provided for the dose rate at surface points or any other off-axis points.

Given the lack of useful experimental data in the literature for off-axis dose distributions for asymmetric plaques, we utilized the information of dose rate at surface points provided in the calibration certificates as reference data to validate our MC models. Our surface dose reference dataset was calculated by averaging equivalent measurement points, for each plaque type, over all calibration certificates received in our institution since 2007, that is, a total of 12 certificates for each plaque type.

**Figure 2:**
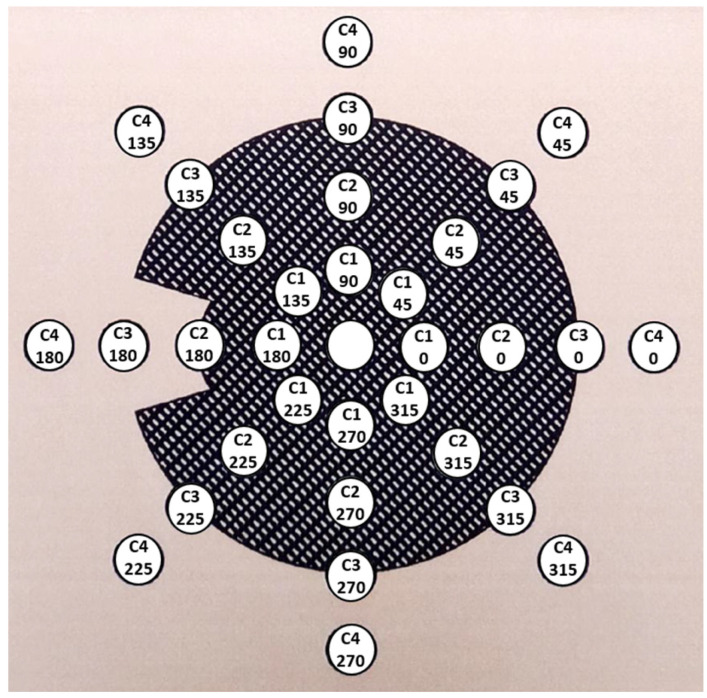
Figure of the distribution of the surface measurement points taken from the calibration certificate of a COC plaque. The relative dose rate value inside each circle has been replaced by the position label adopted in this work, where C1, C2, C3, and C4 refer to the four different concentric circles, and the number below indicates the angular position in each circle.

Information about the coordinates of the surface measurement points was provided by BEBIG after request. Recently, this information has been included in the calibration certificates. The measurement points are placed at 33 positions distributed on 4 concentric circles, each at 1.0 mm distance from the plaque’s inner surface. In each circle, 8 measurement points are distributed with a 45° angular separation. We labeled the points as shown in Figure 2, which depicts a scheme of the positions of the surface measurement points.

Table 1 shows the relative surface dose values of the different measurement points for each type of asymmetric plaque resulting from the average of the values reported in the calibration certificates of 12 plaque lots received in our institution for the last 14 years. The values of the angles 225°, 270°, and 315° are not reported as they were averaged and included with their symmetrically equivalent position corresponding to the angles 135°, 90°, and 45°, respectively.

### 2.3. Monte Carlo Simulations

The *EyeMC* ocular plaque calculation system, described in our previous work by Miras et al. [16], was used to perform the MC simulations of the plaques. It uses a parameterizable MC model for ophthalmic applicators, meaning that the geometric parameters defining a plaque type are read from an external configuration file, thus being easily modifiable. The MC code implemented in *EyeMC* is a modified version of PENELOPE+penEasy, intended to improve the efficiency by forcing the emitting positions to be homogeneously distributed across the active layer using a sampling process. We ran our *EyeMC* simulations upon *CloudMC* [21,22], taking advantage of the computational power provided by Microsoft Azure’s cloud computing services.

For each asymmetric plaque type, we ran a simulation using the official (vendor-reported) values of the geometric parameters and several simulations using the modified geometry and varying the value of the inactive rim parameter. The simulated dose distributions in water were compared to experimental dosimetric data from three different sources: (i) Surface dose reference data obtained from the applicator calibration certificates, (ii) radiochromic film measurements, and (iii) experimental data published in other works. The dose distributions for all simulations were scored in 0.25-mm side cubic voxels and the cutoff energies set for photons and electrons were 4 keV and 100 keV, respectively. For each run, 5 × 10^8^ initial particles were simulated, providing a statistical uncertainty around 1% (k = 2) at 1 mm depth.

In Miras et al. [16], we used the results from Hermida-López et al. [13], which were based on the same MC codes PENELOPE+penEasy, to validate our MC models for the Ru applicators. In this work, to strengthen the validation of our models with an independent MC code, the TOPAS v 3.6.p1 [23,24] MC toolkit was used to simulate the CCD plaque type and the results were compared with those from the *EyeMC* simulation. TOPAS is a medical physics-oriented wrapper and extender for the general-purpose MC code Geant4 [25]. The *Livermore* electromagnetic physics constructor from Geant4 was employed, which provides higher accuracy at tracking low-energy electrons [26], which simulates electrons with kinetic energies of up to 10 eV. Dose was scored across a grid of 0.25-mm voxel size after 10^5^ histories (primary particles generated).

### 2.4. Clinical Cases

In our previous work [16], we used *EyeMC* to calculate a diverse sample of real clinical cases treated in our institution. This sample included cases with different types of plaque (COMS plaques loaded with I-125 seeds and Ru/Rh symmetric and notched plaques) tumor sizes and locations. The dose to tumor and to the critical eye structures was compared with the results of the Plaque Simulator calculations. In this work, the same four clinical cases from our previous work that used asymmetric applicators were re-simulated using the modified geometry to quantify its influence on the dose to critical eye structures.

### 2.5. Film Measurements

**Figure 3:**
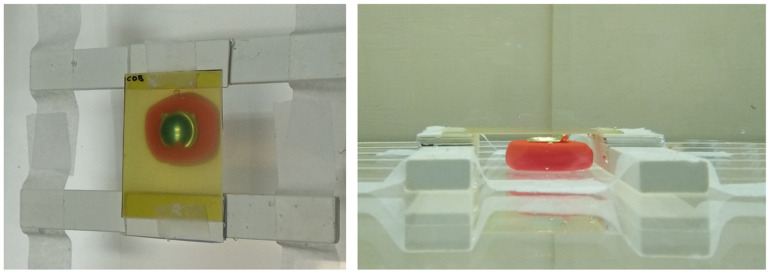
Zenithal (**left**) and lateral (**right**) pictures of the experimental setup designed to measure 2D dose distributions in water for Ru ophthalmic applicators. A COB plaque is set up in this figure.

We adopted the practical method described by Hermida-López et al. [27] distributions in water produced by Ru ophthalmic applicators using EBT3 radiochromic films. A mold was made for each type of asymmetric plaque using dummy plaques and polymer clay. The mold with the plaque is to be placed inside a small water tank. A structure built with Solid Water^®^ (Sun Nuclear Corp. Melbourne, FL, USA) pieces allows us to locate a radiochromic film piece over the plaque in a plane perpendicular to its CAX. Figure 3 shows a zenithal and a lateral view of the experimental setup.

Two-dimensional dose distributions perpendicular to the plaque axis were measured for each plaque type using the experimental setup described. Details on the film-to-plaque distance, irradiation time, and estimated dose to the center of the film for each plaque type are provided in Table 2. The irradiated film pieces were scanned 24 h after irradiation on a 10000XL EPSON flatbed scanner in transmission mode, 16 bits per color channel and 150 dpi resolution. Each film cut was scanned at the center of the scanner together with an unexposed control film cut.

A calibration of the film lot was carried out the same day as the plaque film measurements. A film was cut into 10 strips and nine of them were irradiated in a Solid Water phantom at 5 cm depth and 100 cm source-to-surface distance with a 6 MV 10 × 10 field in a VERSA-HD (Elekta Medical Systems, Stockholm, Sweden) linear accelerator. The strips were irradiated with doses (in Gy): 0.47, 0.95, 1.90, 3.79, 6.64, 9.48, 14.22, 18.96, and 28.44. After 24 h irradiation, the film pieces were scanned following the same scanning protocol described before. The scanned RGB image was used to extract the mean pixel value of the red channel measured at the center of each strip. The corresponding net optical density was then calculated as $nod=log_{10}\left(\frac{PV_{0}}{PV_{exp}}\right)$, where $PV_{0}$ is the mean pixel value of the unexposed strip and $PV_{exp}\;$ the mean pixel value of the exposed film. A calibration function with the form $nod=a\cdot D+b\cdot D^{n}$ was fitted to the *nod-D* pair values [28]. Energy independence can be considered for the radiation sources and range energies involved in this study, which allows us to use a 6 MV calibration for films irradiated with Ru-106 [29]. The images of the film pieces irradiated with the ophthalmic applicators were first processed by applying a 5 × 5 median filter and secondly converted to nod values using the mean pixel value of the unexposed control film cut as $PV_{0}$. Finally, the calibration function was applied, thus generating the corresponding dose planes.

## 3. Results

### 3.1. TOPAS vs. PenEasy Simulations

**Figure 4:**
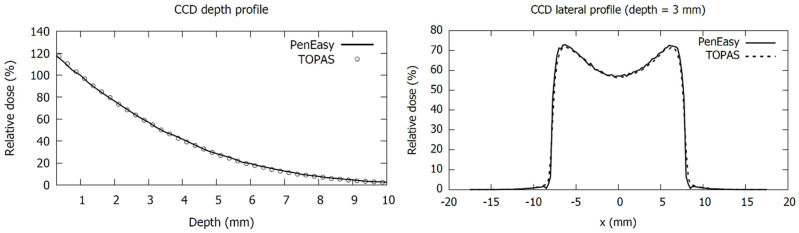
Comparison of central-axis depth profiles (**left panel**) and 3 mm depth lateral profiles (**right panel**) for a CCD plaque simulated with TOPAS and PenEasy. Dose values are normalized to 1 mm depth.

We found excellent agreement between PenEasy and TOPAS MC simulations for plaque CCD, as is shown in the depth and lateral profiles presented in Figure 4. We obtained a mean relative deviation of 2.9% in the range 0 to 10 mm depth, which is within the mean statistical uncertainty in the same range. This result strengthens the validation of our Ru plaque MC models as (i) PenEasy and TOPAS are based in different MC codes using different physics models for the electromagnetic interactions of electrons, PENELOPE and Geant4, respectively, and (ii) the source and geometric models in each MC application were designed by different researchers.

### 3.2. Geometric Parameters

**Figure 5:**
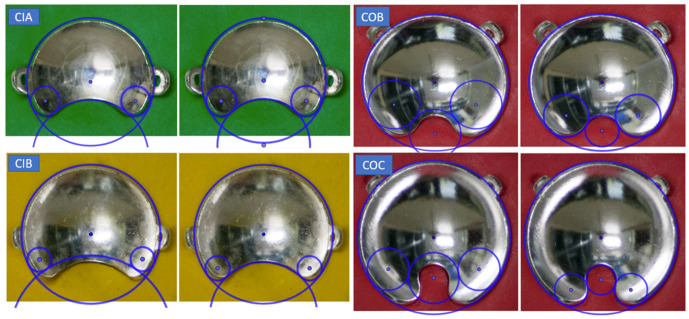
Pairs of pictures of the asymmetric plaques in their molds with the superposition of the geometric forms, generated with the Geogebra program, used to parameterize the cutout. For each plaque type, the official (**left image**) and modified parameters (**right image**) are presented in adjacent images.

Figure 5 shows how the tuned-up values for the geometric parameters fit, in general, better than the official values to the real shape of the notch. The plaque showing the smallest discrepancy with the official parameters is the CIA, while the largest discrepancies are obtained for the COC. The optimal values found for the notch parameters, *a*, *r_1_*, and *r_2_* for each plaque are reported in Table 3.

### 3.3. EyeMC vs. Reference Data

The inactive rim size value in the modified geometries was optimized by minimizing the differences to the MC simulations in the surface points belonging to circle 3 (the closest to the plaque edge), where the rim parameter has a greater effect. The optimal rim size values found are reported in Table 3.

MC dose distributions from *EyeMC* calculations were normalized to the CAX point at 1 mm depth for comparison with reference data. As Table 4 shows, MC calculations using official geometric parameters failed to estimate the dose at surface points. Most of these points presented deviations much larger than the standard deviations of the reference values, with average difference at about 9% and maximum difference going up to 40%. In contrast, the calculations with the modified geometry agreed well with the reference data, with only a few points having deviations slightly larger than the standard deviations but average difference of 2%.

As we did in our previous work [16], depth dose points for each plaque type were compared with the reference data provided by the manufacturer in the applicator’s user manual. In this case, the normalization point is at 2 mm depth in the CAX. The differences presented in Table 5 are slightly larger for the modified geometries with respect to the official ones, although they are contained within the combined uncertainties of the reference data measurements and the MC calculations.

### 3.4. MC vs. Published Data

**Figure 6:**
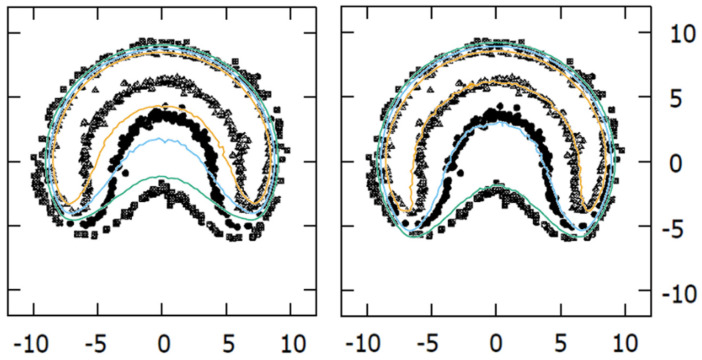
Comparison of isodoses (20%, 30%, and 40% in green, blue, and orange color lines, respectively) at a 4.5 mm depth plane obtained with our MC calculations for the CIB plaque, overlaid on the Taccini et al. 1997 [8] radiochromic film measurements with the official (**left panel**) and modified (**right panel**) geometries.

We evaluated our experimental results against those of Taccini et al. [8] and Trichter et al. [10], as they presented experimental results for asymmetric plaques in a format that allowed comparison. We obtained notable agreement between the results from Taccini et al. [8] and our MC calculations using the modified geometry. As shown in Figure 6, at a 4.5 mm depth, the dose plane from the MC simulation using the official geometry does not match results from Taccini et al. [8] as well as the modified geometry.

**Figure 7:**
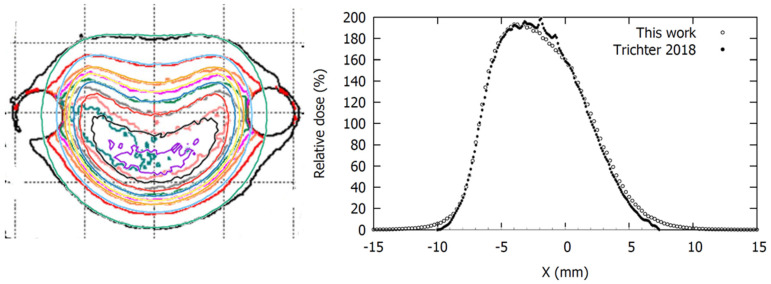
Comparison of isodose distributions (**left panel**) and lateral profiles (**right panel**) at 3.505 mm depth obtained with our MC simulations for the CIA plaque with modified geometry overlaid on the radiochromic film measurements from Trichter 2018 [10]. Isodose levels represented in the left panel are 190, 180, 160, 140, 120, 100, 60, and 20 cGy/h.

The comparison of the CIA calculations to the film measurements presented by Trichter et al. [10] also showed better agreement for the modified geometric model. In the left panel of Figure 7 our isodose distribution is overlaid on the one presented by Trichter et al. [10]. A good coincidence of the isodose lines was observed, except in the regions close to the lateral circles, which correspond to the cutouts in the film made to be inserted into the phantom. It is well known that cutting the films produces stress within the active layer close to the edge that affects its response [30] so the experimental values in these bordering regions cannot be considered. The lateral profiles represented on the right panel correspond to the central vertical axis of the dose planes from the left panel.

### 3.5. Film Measurements

Despite the precautions taken when placing the plaques in the polymer clay mold, the plaques were positioned with some degree of tilt. That was the case for the measurements for the COB and the COC plaques, for which a 1.5° tilt angle perpendicular to the notch direction and a 3° tilt angle in the notch direction were estimated, respectively. To correct for this effect, the estimated tilt was applied to the planar dose distributions generated from our MC simulations for both the official and modified geometries at the plane corresponding to the film measurement.

**Figure 8:**
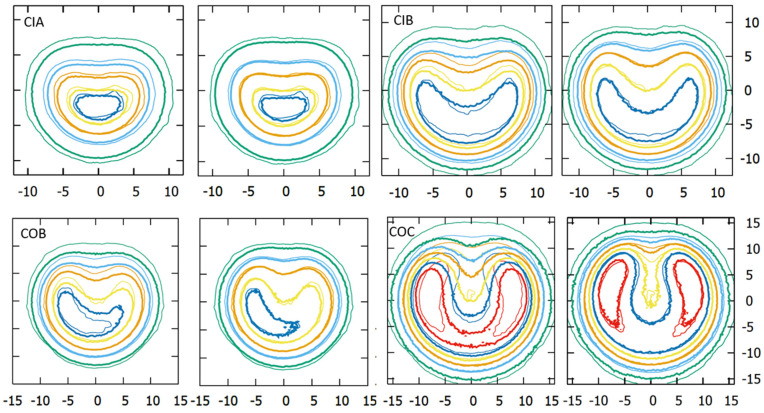
Isodose distribution comparisons, at depths reported in Table 2, for each plaque type between film measurements (thin lines) and MC calculations (thick lines) using the official (**left image**) and the modified geometry (**right image**). Dose planes are normalized to the dose of the central point (100%). Isodose levels represented for each plaque measurement are: 110%, 100%, 70%, 40%, and 10% for the CIA, 130%, 100%, 60%, 30%, and 10% for the CIB, 120%, 100%, 60%, 30%, and 10% for the COB and 200%, 140%, 100%, 60%, 30%, and 10% for the COC plaque. COB dose planes have a 1.5° tilt perpendicular to the notch direction. COC dose planes have a 3° tilt in the notch direction. *x*/*y* axis units are millimeters.

As shown in Figure 8, the dose planes from MC calculations using the modified geometry had a high agreement with the film measurements, while those from the official geometry showed large deviations in the region of the notch. Agreement worsened in the low dose region, where MC predicted lower dose values than the film measurement.

### 3.6. Clinical Cases

**Figure 9:**
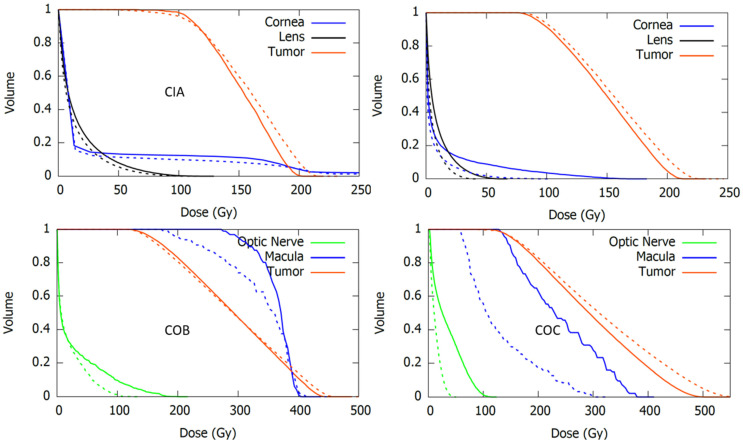
Dose-volume histogram curves for the tumor and the critical structures resulting from real clinical cases calculated with *EyeMC* using the official (dotted lines) and the modified (continuous lines) geometric models for the asymmetric applicators CIA (**upper-left**), CIB (**upper-right**), COB (**bottom-left**), and COC (**bottom-right**).

The results of the recalculated clinical cases using the modified geometry models are presented and compared with those using the official geometry in Table 6 and Figure 9. Table 6 reports the mean dose to the different structures of our patient-specific eye model, while Figure 9 shows the corresponding dose-volume histogram curves.

In general, an increase in the dose to critical structures was observed for calculations using the modified geometries. The largest discrepancies with respect to the official geometry were obtained in the dose to optic nerve and macula for the COC case. Large differences were also observed in dose to lens and cornea for the CIB case. Plaque Simulator results, also included in Table 6, were closer to the modified geometry calculations.

## 4. Discussion

The use of three independent sources of experimental data to validate our results strengthens the thesis of this work. Nonetheless, our aim was not to propose a new definite value set for the geometric parameters but rather to point out the need to improve the geometric models used to simulate asymmetric plaques. In order to facilitate comparison with our results, we have included Table S1 as Supplementary Material containing lateral profiles at different depths for each plaque type extracted from the MC calculations with the modified geometry.

BEBIG reported the absolute dose measurement of the central point with ±20% (2σ) uncertainty until 2019 and ±11% since 2020, but no uncertainties are given for the relative dose rates measured at the surface points. The statistical variability (standard deviation reported in Table 4) from different calibration certificates can be considered as an estimation of the uncertainty (1σ) combining both the uncertainty of the measurement and the inhomogeneities in the distribution of the radioactive isotope across the active layer. According to Zaragoza et al. [31], the latter can reach values up to 25%. It is worth noting that, as pointed out by Hansen et al. [32], BEBIG does not subtract Cerenkov contributions during Ru plaque measurements, which has been found to be dependent on the dose rate reaching the optical fiber and the orientation of the scintillator detector [33]. As the surface dose rate values reported in the certificate are values relative to the central point, they are only affected by relative differences of the Cerenkov signal between different measurement points. Eichmann et al. [33] observed an increasing Cerenkov ratio in the scintillator positions beyond the active surface of the plaque in the experimental setup for surface dose measurements, which is the case for the points in the fourth circle according to the notation adopted in this work. Using the results of Eichmann et al., we estimated the Cerenkov correction for the point (C4, 0°) of the CIB plaque (see Figure 2). However, the difference between the uncorrected value of 5.2% and the corrected value of 4.9% seems not significant considering our estimated uncertainty of 0.7%, obtained from combining the uncertainties reported in Table 4 for the C4 points of the CIB plaque.

In order to find the cause of the isodose disagreement between film measurements and MC calculations in the low-dose region, some simulations were carried out, including the Ru/Rh-106 gamma spectrum, obtaining results similar to those of Hermida-López et al. [34], who reported an insignificant contribution of the gamma spectrum for clinical depths (below 10 cm). Besides gamma contribution, there are at least two other factors that may have a significant impact on the isodose distributions: (a) The measurement depth as to film measurements and (b) the inactive rim size parameter as to MC simulations. For the former, we determined the distance from the film to the central point of the plaque’s inner face from the heights, measured with a caliper, of the different elements conforming to the experimental setup: The plaque, the mold, and the Solid Water structure. This procedure yielded an estimated uncertainty of the film to plaque distances of 1 mm, which is a relatively high value considering the small dimensions of the experimental setup and the steep dose gradients of the Ru applicators. For the latter, no information regarding the uncertainty of the size of the inactive rim was available. We determined its optimal value for each plaque type by minimizing the differences between the MC calculations and the reference data at the points close to the edge of the plaque. However, this method does not necessarily approach the actual inactive rim size. As shown, variations of tenths of millimeters produce appreciable differences in the isodose distributions. Therefore, it seems reasonable to assume that the combined uncertainty of the measurement depth and the inactive rim size explain the differences observed in the low-dose region of the dose planes represented in Figure 8.

The normalization adopted in Figure 6 consisted in estimating the central relative dose value from dose plane by Taccini et al. [8] and assigning this value to the corresponding point of our MC generated dose planes. Results from Taccini et al. [8] lacked a clear normalization procedure to represent the isodose lines for the CIB plaque. In the figure where they presented a depth dose curve measured for a CCA plaque, the data was normalized to a CAX point at 2 mm depth. Applying this normalization to the CIB plaque calculations, as Hermida-López et al. did in their work [13], produces a relative dose value in the center of the dose plane around 60%, while the corresponding value in the figure by Taccini is between the values of 20% and 30%. Hermida-López et al. [13] attributed the large differences in the dose planes to the possibility that Taccini’s dose plane corresponded to another applicator such as the COB. In this work we assumed that the plaque type reported by Taccini et al. [8] was correct, but they used a different normalization.

Clinical cases show how much variations in the geometry of plaques can affect the estimated dose to critical structures. In general, calculations using the modified geometry resulted in higher doses to the structures close to the notch. Of note, in the resimulation of the clinical cases with the modified plaque geometries, the plaque position coordinates on the eye were not changed. However, the positioning of the plaque on the eye is often determined by adjusting the notch to the critical organ that is to be preserved. Thus, if the notch depth parameter is changed, it would affect the plaque position coordinates.

**Figure 10:**
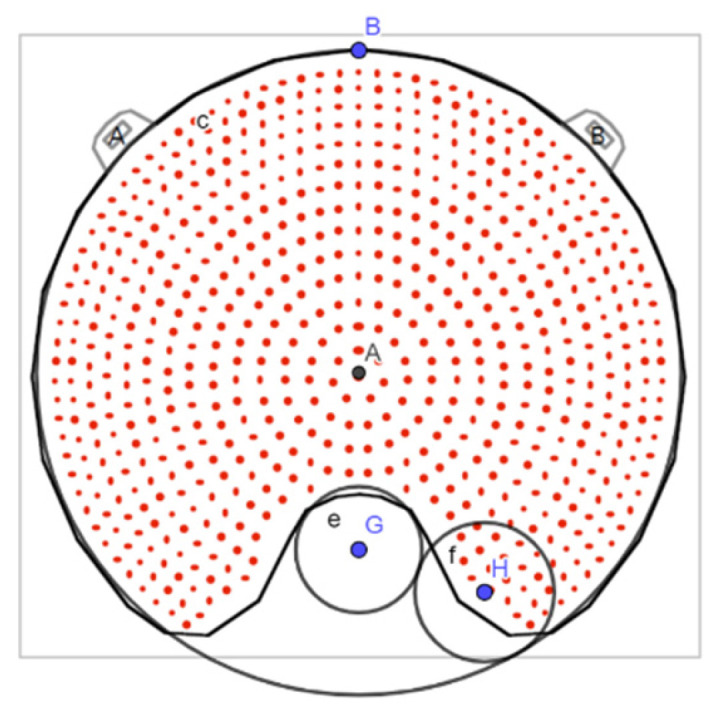
Plaque Simulator diagram of the COC plaque with the superposition of the circles that parameterize the cutout with the parameter values corresponding to the modified geometry.

The fact that Plaque Simulator results for the clinical cases are closer to the MC calculations using the modified geometries might suggest that the geometric models implemented in the planning system differ from the geometric information provided in the applicator user manual. Indeed, when exchanging the pictures of the plaques in Figure 5 by the plaque diagrams from the Plaque Simulator, it is observed that the superimposed circles corresponding to the modified parameter values are better adjusted, although not completely, to the plaques diagram. An example of this for the COC plaque is shown in Figure 10.

## 5. Conclusions

Personalized eye dosimetry is required for an optimal treatment planning able to deliver the prescription dose to the tumor and OAR sparing. Given the mentioned problems associated with the experimental measurements of Ru ocular plaques, MC techniques remain the best alternative tool for providing a dosimetric characterization precise enough for personalized ocular brachytherapy. However, an essential condition to build good MC models is having a precise characterization of the radiation source geometry. Our results indicate that the geometric parameterization of the asymmetric Ru plaques proposed by the manufacturer is adequate for modeling the geometry of the cutout region, but the geometric parameter values provided for each plaque type do not necessarily fit properly to their real shape. Therefore, its implementation in MC models may lead to wrong results. The graphical method described in this work allowed us to find new values for the geometric parameters that fit better to the real geometry. MC simulations of the notched plaques using these modified geometries produced results agreeing better with the experimental data published in the literature, the surface dose point measurements reported in the plaques calibration certificates, and radiochromic film dose measurements performed for this work.

Finally, we have shown that variations on the values of the geometric parameters may have an important effect on the estimated dose to critical structures in real ocular plaque treatments. A good geometric characterization of the asymmetric applicators is thus essential for accurate positioning and dosimetry in this kind of personalized treatment.

## Figures and Tables

**Table 1:** Average relative surface dose values for the measurement points reported in the calibration certificates obtained from certificates from 12 different plaque lots. Standard deviation values given in brackets. Values are given in percentage and normalized to the CAX point.

Circle	Angle	CIA	CIB	COB	COC
1	0°	104.3 (3.5)	99.7 (4.1)	97.8 (4.0)	94.2 (6.3)
1	45°	104.3 (2.9)	99.7 (2.8)	98.0 (3.0)	96.8 (5.1)
1	90°	101.1 (1.5)	100.3 (2.5)	99.4 (3.2)	98.7 (2.7)
1	135°	72.4 (4.6)	91.9 (4.2)	98.0 (4.7)	91.0 (7.0)
1	180°	44.3 (4.4)	62.8 (6.8)	82.5 (7.4)	32.3 (4.9)
2	0°	99.6 (7.2)	98.0 (6.4)	97.3 (6.2)	93.3 (8.8)
2	45°	102.1 (4.9)	99.2 (3.7)	96.4 (5.2)	94.4 (5.7)
2	90°	99.1 (3.8)	99.0 (5.5)	97.0 (4.7)	97.2 (4.2)
2	135°	41.3 (4.0)	63.3 (7.1)	94.1 (6.7)	97.8 (5.9)
2	180°	12.1 (1.6)	10.5 (1.6)	19.4 (2.2)	10.3 (1.4)
3	0°	59.3 (8.9)	48.9 (6.2)	44.3 (9.2)	34.1 (8.2)
3	45°	64.1 (6.5)	56.5 (6.6)	51.7 (6.8)	43.7 (11.7)
3	90°	62.2 (6.9)	61.3 (6.6)	58.8 (6.9)	48.8 (6.9)
3	135°	17.5 (1.8)	29.6 (5.1)	55.0 (9.8)	52.5 (6.3)
3	180°	4.1 (0.5)	3.3 (1.5)	5.6 (0.8)	3.4 (0.7)
4	0°	10.9 (1.3)	5.2 (0.7)	5.5 (1.2)	2.0 (0.4)
4	45°	14.7 (1.8)	5.7 (0.8)	6.4 (0.7)	2.4 (0.6)
4	90°	11.7 (1.1)	6.5 (0.7)	6.8 (0.5)	2.9 (0.4)
4	135°	4.8 (0.6)	3.5 (0.6)	5.7 (0.9)	2.8 (0.4)
4	180°	1.7 (0.5)	0.9 (0.3)	1.7 (0.5)	0.9 (0.3)

**Table 2:** Film–plaque distance (FPD), irradiation time (T), and estimated dose (D) to the center of the film piece for each plaque measurement.

Plaque	FPD (mm)	T (h)	D (Gy)
COB	7.0	4.73	3.97
COC	9.4	17.75	5.04
CIA	5.4	2.55	2.98
CIB	6.4	3.68	3.45

**Table 3:** Geometric parameters of the asymmetric plaques with the modified values derived from this work, with *D* being the plaque diameter, *R* the curvature radius, *s* the size of the inactive rim, *a* the notch depth, *r*_1_ the notch curvature radius, and *r*_2_ the curvature radius of the notch edges. All numeric values are expressed in mm. For the modified parameters, their official values are given in brackets for comparison.

Plaque Type	*D*	*R*	*s*	*a*	*r*_1_	*r*_2_
CIA	15.3	12	0.4 (0.75)	5.40 (5.5)	5.70 (7.0)	1.86 (1.75)
CIB	20.2	12	0.6 (0.75)	6.10 (7.0)	7.80 (11.75)	1.78 (1.75)
COB	19.8	12	1.0	5.18 (6.0)	2.28 (3.5)	3.055 (3.5)
COC	25.4	14	1.0	8.21 (10.2)	2.48 (4.2)	2.742 (3.5)

**Table 4:** Surface points dose differences, expressed as a percentage, between the reference data, reported in Table 1 and our MC calculations using the official and modified geometries. Difference values superior to the standard deviations reported in Table 1 are highlighted in bold.

Circle	Angle	CIA		CIB		COB		COC	
		Official (%)	Modified (%)	Official (%)	Modified (%)	Official (%)	Modified (%)	Official (%)	Modified (%)
1	0°	**6.2**	2.7	**8.0**	2.2	**5.2**	3.3	**18.4**	**7.2**
1	45°	**5.0**	1.3	**7.0**	2.2	**5.0**	2.8	**16.4**	4.1
1	90°	0.4	−0.3	2.1	−0.6	**1.0**	0.1	**9.2**	1.1
1	135°	**−10.9**	3.3	**−24.8**	−3.7	**−9.9**	−3.2	**−41.7**	−1.2
1	180°	**−9.2**	2.3	**−31.3**	−0.5	**−27.0**	1.2	**−18.4**	**6.8**
2	0°	3.1	0.8	4.7	−0.7	−0.8	−2.5	**17.2**	3.3
2	45°	−0.2	−2.5	2.2	−2.2	0.5	−1.7	**15.5**	3.0
2	90°	−2.4	−3.2	−0.5	−3.4	−1.9	−2.8	**11.8**	−0.4
2	135°	**−13.7**	1.6	**−40.1**	−4.1	**−22.7**	**−7.7**	**−28.3**	**−7.2**
2	180°	**−2.7**	0.1	**−4.8**	−0.6	**−8.5**	0.1	**−7.0**	−0.2
3	0°	−8.4	1.5	3.5	**8.4**	5.4	9.1	**20.8**	**13.2**
3	45°	**−13.4**	−3.7	−4.3	0.2	−1.8	2.2	11.6	3.4
3	90°	**−14.6**	−3.6	**−10.8**	−5.3	**−9.4**	−5.5	6.1	−1.7
3	135°	**−6.7**	0.8	**−21.9**	−3.7	**−27.4**	−7.1	**−29.1**	**−7.6**
3	180°	**−0.7**	0.1	**−1.9**	−0.8	**−2.6**	0.1	**−2.7**	0.2
4	0°	**−1.8**	0.1	0.4	**1.1**	0.4	0.8	**1.3**	**0.9**
4	45°	**−5.7**	**−3.8**	0.0	0.5	−0.4	−0.3	**0.8**	0.4
4	90°	**−3.5**	**−1.5**	**−1.3**	−0.5	**−1.1**	**−0.6**	0.3	−0.1
4	135°	**−1.7**	−0.3	**−1.9**	−0.4	**−2.4**	−0.8	**−1.1**	−0.4
4	180°	−0.5	−0.2	**−0.6**	−0.3	**−0.9**	−0.2	**−0.8**	−0.3

**Table 5:** Relative dose difference, expressed in percentage, in the central axis between the reference data provided by BEBIG and our calculations using the official and modified geometric models. Positive/negative values indicate that the MC value is higher/lower than the reference value. The normalization was set to the CAX point at 2 mm depth.

Depth (mm)	CIA		CIB		COB		COC	
	Official	Modified	Official	Modified	Official	Modified	Official	Modified
1	−1.56	−4.79	1.08	−2.22	−0.07	−2.46	2.19	−2.65
2	0.00	0.00	0.00	0.00	0.00	0.00	0.00	0.00
3	0.75	1.54	−0.32	0.83	0.35	0.84	0.43	1.84
4	1.25	2.18	0.97	2.50	1.10	1.95	1.84	2.72
5	0.76	1.47	0.75	1.84	1.28	1.89	1.56	2.12
6	0.45	1.13	0.39	1.27	0.91	1.31	0.95	1.42
7	0.20	0.67	0.30	0.81	0.55	0.77	0.67	0.87
8	0.04	0.38	0.22	0.64	0.15	0.53	0.34	0.55
9	0.03	0.25	−0.01	0.32	0.17	0.31	0.16	0.39
10	0.00	0.16	0.00	0.22	0.00	0.14	0.00	−0.08

**Table 6:** Dose at apex and mean doses to the tumor and critical structures, in Grays, for the real clinical cases from Miras et al. [16], calculated with the Plaque Simulator (PS) and with *EyeMC* using the official and modified (Mod.) plaque geometries.

Case ID	Patient 2			Patient 3			Patient 4			Patient 5		
Plaque	CIA			CIB			COB			COC		
	Official	Mod.	PS	Official	Mod.	PS	Official	Mod.	PS	Official	Mod.	PS
Apex	82.1	83.3	85.0	84.1	86.0	85.0	80.1	81.4	85.0	89.6	89.7	85.0
Tumor	156.4	151.4	156.4	153.9	147.5	140.2	289.0	289.1	301.8	315.3	294.5	259.5
Sclera	12.7	13.4	12.3	24.9	25.8	23.4	67.1	69.6	67.2	112.9	111.0	89.4
Macula	-	-	-	-	-	-	334.1	361.2	372.7	128.9	238.1	208.6
Optic nerve	-	-	-	-	-	-	18.9	27.8	59.6	12.7	33.7	28.4
Retina	7.6	8.3	8.3	13.2	14.6	11.9	33.1	33.9	32.3	48.5	49.8	41.6
Lens	13.1	15.8	21.3	5.3	8.9	10.6	-	-	-	-	-	-
Cornea	25.5	33.0	30.7	5.3	12.3	16.6	-	-	-	-	-	-

## Data Availability

The data that support the findings of this study are available from the corresponding author upon reasonable request.

## Supplementary Materials

The following supporting information can be downloaded at: https://www.mdpi.com/article/10.3390/jpm12050723/s1, Table S1: Dosimetric data set for Ru asymmetric plaques.
