# Supplementary material for: Modified Geometry of 106Ru Asymmetric Eye Plaques to Improve Dosimetric Calculations in Ophthalmic Brachytherapy
Source: J Pers Med. 2022 Apr 29;12(5):723. doi: 10.3390/jpm12050723 (PMC9144752; doi:10.3390/jpm12050723)
Supplement: Supplementary file 1 [file jpm-12-00723-s001.zip › jpm-1674725-supplementary.pdf]

Supplementary material: Dosimetric data set for $^{106}Ru/^{106}Rh$ notched plaques with modified geometric models.

Table S1. Lateral relative dose profiles at several depths for the ophthalmic applicators CIA, CIB, COB and COC calculated with the modified geometric models.

Depth (mm)	Lateral distance from the CAX (mm)																		
CIA X	0	1.5	2.5	3.5	4.5	5.5	6.5	7.5	8.5	9.5									
1	100.0	104.5	109.7	116.6															
2	71.8	74.8	79.6	84.6	91.3	98.1													
3	50.6	52.0	54.8	58.2	59.7	56.7	38.7	12.1	3.8	1.6									
4	34.8	35.8	37.1	37.7	37.2	32.3	22.9	11.7	5.1	2.2									
6	14.8	14.9	15.0	14.8	13.5	11.7	8.8	5.9	3.4	1.9									
8	5.4	5.6	5.4	5.2	4.6	4.0	3.1	2.3	1.6	1.0									
10	1.7	1.7	1.6	1.6	1.5	1.2	1.0	0.7	0.5	0.4									
CIA Y	-9.5	-8.5	-7.5	-6.5	-5.5	-4.5	-3.5	-2.5	-1.5	0	1.5	2.5	3.5	4.5	5.5	6.5	7.5	8.5	9.5
1							120.8	115	110	100	72.5	33.3	14.5	7.3	3.9	2.1	1.2	0.6	0.3
2					100.3	96.1	91.2	85.7	81.2	71.8	51.8	33.5	18.3	9.7	5.3	3	1.5	0.8	0.4
3	1.8	4.3	12.9	40.5	58.6	64.0	63.3	61.4	58.5	50.6	37.8	26.8	17.4	10.2	5.8	3.1	1.7	0.9	0.4
4	2.4	5.4	12.3	24.4	35.2	40.3	42.1	41.5	39.3	34.8	26.5	20	13.8	9	5.3	3	1.7	0.9	0.4
6	2.1	3.9	6.5	9.9	13.0	15.2	17.0	17.2	16.8	14.8	11.8	9.5	7	4.9	3.1	2	1.2	0.6	0.3
8	1.1	1.8	2.6	3.7	4.7	5.5	5.8	6.3	6.1	5.4	4.5	3.7	2.8	2.1	1.4	0.9	0.6	0.3	0.2
10	0.4	0.6	0.9	1.2	1.4	1.7	1.9	1.9	1.9	1.7	1.5	1.1	0.9	0.6	0.5	0.3	0.2	0.1	0.1
CIB X	0	1.5	2.5	3.5	4.5	5.5	6.5	7.5	8.5	9.5	10.5	11.5							
1	100.0	101.6	108.0	114.0															
2	75.4	77.6	82.2	88.9	97.1	108.2													
3	56.3	57.8	61.9	66.7	74.7	83.2	94.1												
4	39.6	42.5	44.5	49.2	54.6	61.0	68.0	75.7	77.8										
6	19.2	20.0	21.3	23.2	25.6	27.2	28.0	26.1	18.7	9.5	4.0	1.6							
8	8.0	8.3	8.8	9.4	10.0	10.3	10.2	8.6	6.5	4.7	2.7	1.4							
10	2.9	2.9	3.1	3.2	3.3	3.3	3.3	2.8	2.3	1.8	1.2	0.7							
CIB Y	-9.5	-8.5	-7.5	-6.5	-5.5	-4.5	-3.5	-2.5	-1.5	0	1.5	2.5	3.5	4.5	5.5	6.5	7.5	8.5	9.5
1							116	110	105	100	95.6	86.5	48.1	13.9	6.1	3.4	1.8	1	0.5
2					110	100	91.5	85.7	80.4	75.4	69.7	58.4	39.6	20.9	10.5	5.4	2.9	1.4	0.7
3				96	85.9	76.9	70.1	65.4	60.5	56.3	49.4	41.7	30.9	19.9	11.4	6.4	3.5	1.8	1
4		78.4	75.9	70.1	62.6	57.2	51.9	48	44.6	39.6	35	29.9	23.4	16.3	10.4	6.2	3.7	2	1.1

6	9.9	19.3	26.2	28.8	28.4	27.2	25.5	23.8	22.2	19.2	16.5	14.1	11.4	9	6.6	4.3	2.8	1.7	0.9
8	4.7	7	9.3	10.7	11.3	10.9	10.4	10	9.3	8	6.8	6	5	4	2.9	2.1	1.4	0.8	0.5
10	1.8	2.4	3	3.5	3.9	4	3.8	3.4	3.2	2.9	2.3	2.1	1.7	1.3	1.1	0.8	0.6	0.4	0.2

COB X	0	1.5	2.5	3.5	4.5	5.5	6.5	7.5	8.5	9.5	10.5	11.5
1	100	103	107	113								
2	76.3	79.1	82.4	88.6	96.1							
3	56.6	58.7	62.5	66.8	72.9	80.3						
4	41.3	42.8	45.2	47.9	52.2	56.5	60.4	58	31.2	2.3	0.9	0.4
6	19.6	20.4	21.2	22.2	23.4	23.5	22.5	18.4	12.4	6.6	2.9	1.2
8	7.9	8.4	8.8	8.9	9	8.7	8	6.5	5	3.3	1.9	1
10	2.7	2.8	2.9	3.1	3.1	2.8	2.7	2.2	1.7	1.2	0.7	0.5

COB Y	-9.5	-8.5	-7.5	-6.5	-5.5	-4.5	-3.5	-2.5	-1.5	0	1.5	2.5	3.5	4.5	5.5	6.5	7.5	8.5	9.5
1							115	108	104	100	98.4	95.6	74.1	28.1	7	3.7	1.9	1.1	0.5
2					106	97.7	90.5	83.9	80.6	76.3	73.5	67.5	52.9	31.5	15.6	8	3.9	2	0.9
3				90	81.3	74.1	67.7	64.2	60.2	56.6	54	48.2	40	28.7	18.1	10.5	5.6	2.8	1.3
4	2.3	31.8	58.7	60.6	57.5	53.6	49.7	47	44.5	41.3	37.6	34.4	29.9	23.2	16.7	10.5	6.3	3.2	1.8
6	6.4	12.8	18.7	23.1	24.7	24.1	23.3	22	21.4	19.6	18.2	16.4	14.4	12.4	9.8	6.9	4.6	2.9	1.6
8	3.2	5.1	7	8.1	9	9.3	9.4	9.1	8.8	7.9	7.2	6.6	5.9	5.2	4.3	3.3	2.2	1.5	0.9
10	1.3	1.9	2.4	2.8	3	3.3	3.2	3.2	2.9	2.7	2.5	2.4	2	1.8	1.4	1.2	0.8	0.6	0.3

COC X	0	1.5	2.5	3.5	4.5	5.5	6.5	7.5	8.5	9.5	10.5	11.5
1	100	101	105	112								
2	76.3	76.7	81.3	86.7	95.3	105						
3	55.5	58.1	61.7	67.1	73.3	82.3	93.1	107				
4	39.9	41.9	45.4	50.3	55	64.1	72.2	84.2	96.2			
6	20.4	21.3	23.5	26.2	29.6	33.8	39.2	45.5	52.9	58.7	62.1	6.2
8	9.3	9.6	10.3	11.9	13.7	15.9	18.7	20.2	21.5	21.2	16.7	9.9
10	3.4	3.5	3.9	4.9	5.5	6.3	7	7.8	7.6	7.1	5.7	4.4

COC Y	-11.5	-10.5	-9.5	-8.5	-7.5	-6.5	-5.5	-4.5	-3.5	-2.5	-1.5	0	1.5	2.5	3.5	4.5	5.5	6.5	7.5	8.5	9.5
1									114	106	103	100	95.4	89.4	58.4	21.6	9.9	5.7	3.4	1.9	1.1
2							107	95.8	89.6	82.5	79.6	76.3	70.6	64.1	47.9	29.7	17.9	11	6.6	3.5	1.8
3					107	93.6	83.3	74.5	68.8	63.8	59.1	55.5	51.3	47.1	38.6	29.4	21.2	14.5	9.6	5.9	3.3
4				97.6	83.5	73.4	64.5	57.2	51.3	47.5	44.7	39.9	38.1	35	30.4	26	20	15.6	11	7.8	4.8

6	6.2	61.6	58.7	53	46.1	39.9	35.1	31.4	26.9	24.3	22.8	20.4	18.8	17.4	17.1	15.7	13.8	12.6	10.4	8.4	5.5
8	10.2	17.1	20.7	22	21	18.6	16.5	14.1	12.7	11.2	10.2	9.3	8.2	8.1	7.9	7.9	7.4	7.1	6.2	5.1	4
10	4.3	5.7	7.2	8	8	7.3	6.8	5.4	5.1	4.5	3.9	3.4	3.1	3	3.2	3	3.1	3	2.9	2.4	2
